# *Naegleria fowleri* Infections: Bridging Clinical Observations and Epidemiological Insights

**DOI:** 10.3390/jcm14020526

**Published:** 2025-01-15

**Authors:** Carmen Rîpă, Roxana Gabriela Cobzaru, Miruna Raluca Rîpă, Alexandra Maștaleru, Andra Oancea, Carmen Marinela Cumpăt, Maria Magdalena Leon

**Affiliations:** 1 Department of Microbiology, Faculty of Medicine, University of Medicine and Pharmacy “Grigore T. Popa”, Universitatii Street No. 16, 700115 Iasi, Romania; ripa.carmen@umfiasi.ro; 2 Department of Preventive Medicine and Interdisciplinarity, University of Medicine and Pharmacy “Grigore T. Popa”, Universitatii Street No. 16, 700115 Iasi, Romania; 3 University of Medicine and Pharmacy “Grigore T. Popa”, Universitătii Street No. 16, 700115 Iasi, Romania; mg-rom-32810@students.umfiasi.ro; 4 Department of Medical Specialties I, University of Medicine and Pharmacy “Grigore T. Popa”, 700115 Iasi, Romania; alexandra.mastaleru@umfiasi.ro (A.M.); andra.oancea@umfiasi.ro (A.O.); maria.leon@umfiasi.ro (M.M.L.); 5 Clinical Rehabilitation Hospital, 700661 Iasi, Romania; marinela.cumpat@umfiasi.ro; 6 Department of Medical Specialties III, University of Medicine and Pharmacy “Grigore T. Popa”, 700115 Iasi, Romania

**Keywords:** *Naegleria fowleri*, case report, contaminated water

## Abstract

Purpose: *Naegleria fowleri* is the main etiologic agent implicated in primary amoebic meningoencephalitis (PAM). It is also known as the brain-eating amoeba because of the severe brain inflammation following infection, with a survival rate of about 5%. This review aims to identify *Naegleria fowleri* infections and evaluate patients’ progression. This literature review emphasizes the importance of rapid diagnosis and treatment of infected patients because only prompt initiation of appropriate therapy can lead to medical success. Compared to other articles of this kind, this one analyzes a large number of reported cases and all the factors that affected patients’ evolution. Materials and methods: Two independent reviewers used “*Naegleria fowleri*” and “case report” as keywords in the Clarivate Analytics—Web of Science literature review, obtaining 163 results. The first evaluation step was article title analysis. The two reviewers determined if the title was relevant to the topic. The first stage removed 34 articles, leaving 129 for the second stage. Full-text articles were evaluated after reading the abstract, and 77 were eliminated. This literature review concluded with 52 articles. Key findings: This review included 52 case report articles, 17 from the USA, eight from India, seven from China, four from Pakistan, two from the UK, and one each from Thailand, Korea, Japan, Italy, Iran, Norway, Turkey, Costa Rica, Zambia, Australia, Taiwan, and Venezuela, and Mexico. This study included 98 patients, with 17 women (17.4%) and 81 men (82.6%). The cases presented in this study show that waiting to start treatment until a diagnosis is confirmed can lead to rapid worsening and bad outcomes, especially since there is currently no drug that works very well as a treatment and the death rate is around 98%. Limitations: The lack of case presentation standardization may lead to incomplete case information in the review since the cases did not follow a writing protocol. The small number of global cases may also lead to misleading generalizations, especially about these patients’ treatment. Due to the small number of cases, there is no uniform sample of patients, making it difficult to determine the exact cause of infection.

## 1. Introduction

*Naegleria fowleri* is the main etiologic agent implicated in primary amoebic meningoencephalitis (PAM) [1]. It is also known as the brain-eating amoeba because of the severe inflammation of the brain following infection, with a survival rate of about 5% [2]. It is most commonly found in water or wet soils and grows very well on cell cultures or various artificial media [1]. Temperatures above 30 °C create a favorable environment for this amoeba, with survival and infection being impossible in the winter season [3]. However, it is not only high temperatures that can be a favorable factor but also the increased amount of suspended organic matter and sediment, which lead to poor water quality and a favorable environment for the development of amoebae [4]. This is the only amoeba species exhibiting three distinct morphologic forms: the trophozoite, flagellate, and cyst [1,5]. *Naegleria fowleri* enters the host’s system via the nasal tract, either by inhalation of dust-containing cysts or by aspiration of water contaminated with trophozoites or cysts. Young people are most often affected, as they are most often exposed to potentially contaminated environments [6]. The clinical course is mostly dramatic, and after an incubation period of 2 to 15 days, the disease has a sudden onset with a fulminant course toward death [7]. Since the officially recorded case of onset, contrary to the evolution of medicine, the number of cases seems to be increasing, with 381 cases reported from 1962 to 2018, originating in 33 countries [3,8]. Between 1965 and 2016, the overall number of reported PAM cases grew by 1.6% every year. During this time, the number of confirmed PAM cases grew by an average of 4.5% yearly [9]. In a 2020 study, it was reported that the worldwide prevalence of Naegleria in various water sources was 26.42%. The highest case rate was found in America, approximately 33.18%, not only due to multiple sources for possible infection, but also due to the large number of studies that have taken place in this territory [10].

This review aims to identify documented cases of *Naegleria fowleri* infection and analyze the evolution of patients since the time of infection. Thus, through this literature review, we want to emphasize the importance of the rapid diagnosis and treatment of infected patients since only the initiation of appropriate therapy as promptly as possible can lead to medical success. Compared to other articles of this sort, this one brings together a large number of reported cases, analyzing in detail all the aspects that had an impact on the evolution of patients infected with Naegleria fowleri.

## 2. Materials and Methods

The literature review was performed by two independent reviewers on the Clarivate Analytics—Web of Science platform using as keywords the association between “*Naegleria fowleri*” and “case report” and 163 results were obtained. The first evaluation step was to analyze the title of the articles. If the title was considered significant for the chosen topic, the two reviewers proceeded to abstract analysis. After the first stage, 34 articles were eliminated, leaving 129 articles for the second stage. After reading the abstract, the next eliminatory stage was the evaluation of full-text articles, and 77 papers were excluded. Finally, 52 articles were included in this literature review.

The inclusion criteria were English language, and case report type articles describing the cases of patients infected with *Naegleria fowleri*, regardless of the evolution they had after confirmation of the diagnosis. All eligible articles were included regardless of the year of publication or the age of patients included in the studies.

Exclusion criteria were articles that were written in full-text in a language other than English, articles that could not be accessed, conference presentations, abstracts, letters to the editor, books, editorial material, proceeding papers or review articles, and articles in which insufficient patient data were identified.

## 3. Results

The general characteristics of the cases included in our study can be observed in Table 1.

The evolution and treatment of the patients included in this review can be observed in Table 2.

**Figure 1:**
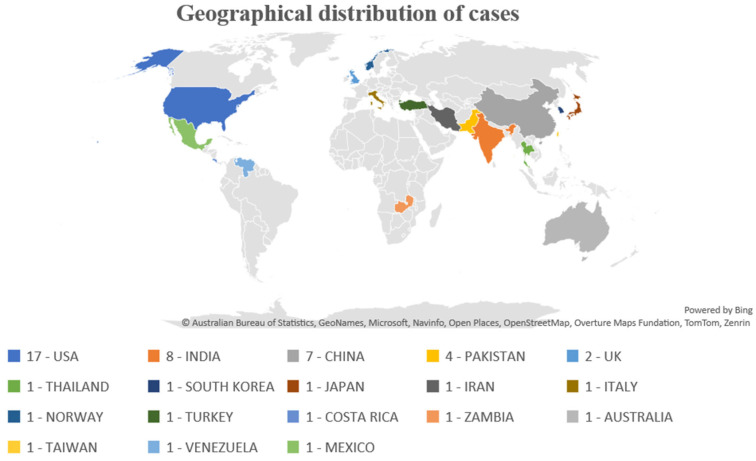
Geographical distribution of cases.

This review included 52 case report articles, 17 from the USA, eight from India, seven from China, four from Pakistan, two from the UK, and one each from Thailand, Korea, Japan, Italy, Iran, Norway, Turkey, Costa Rica, Zambia, Australia, Taiwan, Venezuela, and Mexico. A total of 99 patients were included, including 17 women (17.17%) and 82 men (82.82%). (Figure 1)

The patients’ ages in the study ranged from 11 days to 75 years. There were seven patients less than 1 year old, 16 patients aged between 2 and 10 years, 19 patients aged between 10 and 20 years, 13 patients aged between 21 and 40 years, and 12 patients older than 40 years. In addition, in two of the studies mentioned in the review, there were 19 and 13 patients, respectively, with a mean age of 28 and 31 ± 15.33 years, respectively. Approximately, the age at which patients are more susceptible to *Naegleria fowleri* infection seems to be 10–40 years.

**Figure 2:**
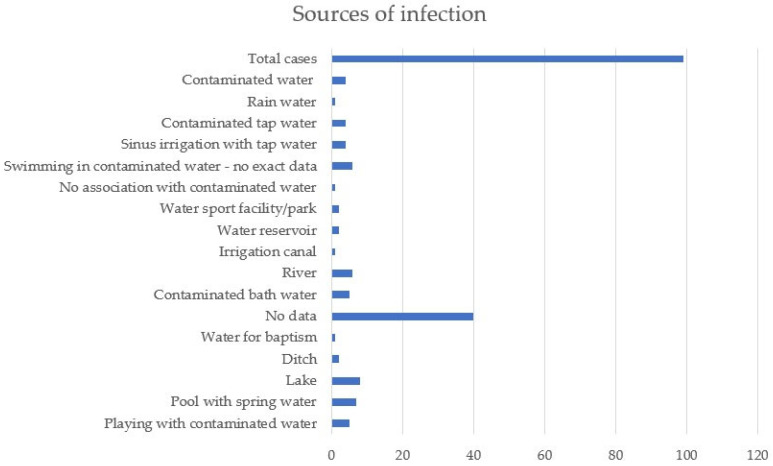
Sources of infection.

Discussing the way of infection, in 40 of the 99 cases, no exact cause or contact with contaminated water could be established, and in only one person, no link between infection and a water source was observed. Five of the patients developed symptoms after playing in areas with contaminated water, seven after swimming in pools that were irrigated with spring water, and eight and six, respectively, after swimming in lakes or rivers. One of the patients under the age of 1 died shortly after baptism, and another after being exposed to water collected after rain. For five people, symptoms started after bathing in possibly contaminated water, and six after swimming in areas where *Naegleria fowleri* was found after water testing. The condition of four of the patients worsened after nasal irrigation with tap water, and of another four after using tap water for various household activities. Other sources of infection were ditch water, canal irrigation, water tanks, or water parks (Figure 2).

The most common symptoms with which patients in the articles included in the review presented to the doctor were fever, headache, and vomiting, symptoms specific for meningeal inflammation. Other less common symptoms were seizures, neck stiffness, fatigue, confusion, and sensory disturbances. In most cases, no exact diagnosis was considered until the results were received. However, patients who presented with symptoms specific to meningeal irritation were given a presumptive diagnosis of viral or bacterial meningitis.

**Figure 3:**
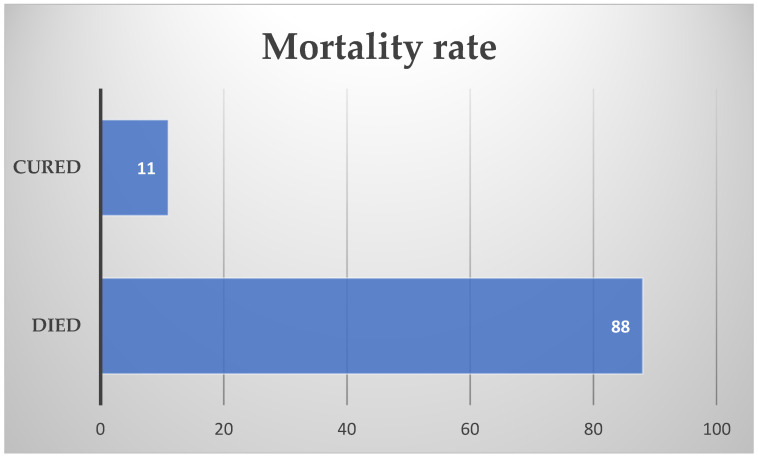
Mortality rate.

Of the 99 patients, only 11 of them survived the *Naegleria fowleri* infection, the mortality rate being 88.88%. (Figure 3) The hospitalization period ranged from 10 h to 4 months. Further discussion on the hospitalization period shows that the most common hospitalization period was 1–5 days (45 patients). Six patients were hospitalized for 5–10 days, 3 for 1–15 days, and 14 for more than 15 days. In one study, the hospitalization period of 13 patients was approximately 6.38 ± 3.15 days. Only one case, a 6-month-old infant, had a fulminant progression to death within 10 h.

Of the patients who survived, the average age was 17.8 years and the average length of hospitalization was 29.5 days. Excluding the only elderly patient in this group (73 years), the mean age for surviving patients was 11.66 years, with a mean number of days hospitalized of 31.67. In terms of patients who did not survive, the average age was about 21 years, with a number of days of hospitalization of around 8.39 days. If we exclude the only patient who was hospitalized for 4 months, the average number of days of hospitalization becomes about 6.25 days. Thus, we could say that the patients who were cured had a much longer period of hospitalization, but also a lower average age, compared to those who did not survive the *Naegleria fowleri* infection.

## 4. Discussion

### 4.1. Prevention

Infection with *Naegleria fowleri* is often fatal, with death occurring in less than 72 h in many patients [50]. As observed in the cases included in this study, it seems that one of the most common causes of infection is contact with contaminated water. Most of the time, patients come in contact with water by swimming in lakes, rivers, or even swimming pools that are not properly sanitized [59]. With modernization, the number of cases among tourists who frequent vacation resorts that include heated swimming pools has increased. For example, a 2005 study evaluating the presence of this amoeba in tourist sites in Thailand found that *Naegleria fowleri* was present in about 40% of the recreational sites sampled [60]. Infection can only be prevented by avoiding these types of recreational areas because, as observed in the cases presented above, most of the hospitalized patients had a history of swimming either in unsanitized swimming ponds or in natural recreational sites such as lakes or rivers. Another method of prevention could be to avoid swallowing or mouth or nostril contact with contaminated water [43].

In the cases presented above, some of the patients came into contact with this amoeba after nasal irrigation with contaminated water; therefore, people practicing this habit should be more careful about the water they use because the olfactory mucosa is one of the entry points of the amoeba [40]. When it comes to cases of infection in newborns, parents should be cautious about the water they use for preparing milk and bathing their babies since they cannot become contaminated by swimming in potentially infectious areas, but only by contact with contaminated water through ingestion or during bathing [51]. For this reason, the diagnosis of PAM is very difficult to consider in this type of patient, thus lowering the success rate of therapy [51]. Among the six cases of children under 1 year of age included in the review, four of them mention contaminated water in which the babies were being bathed as the cause of infection.

Those most susceptible to infection with this amoeba are young people with good immunity, especially young men because they are most often involved in water recreational activities in the warm season [21,31]. As noted in our review, the average hospitalization period until patients most often progress to fatality is about 5–10 days from the onset of symptoms [31]. However the infection rate is quite low, and the incidence of the disease is very low, because rarely do people who come in contact with a potential source of infection develop the disease [47].

### 4.2. Diagnosis

For patients to have a favorable outcome, therapy should be started as soon as possible; for this reason, physicians should be much better informed about this infection and its signs and take it into account when patients are presented in the emergency room [22]. PAM is clinically indistinguishable from classic bacterial meningitis, which is why the amoebic etiology should be suspected whenever the presence of a pathogenic bacterium cannot be detected in the CSF. Criteria that could guide the physician toward the diagnosis of this infection could be young age, recent activity in the aquatic environment, or contact with various water sources, especially heated swimming pools, rivers, lakes, or stagnant water [3,7,31]. Even when considering possible Naegleria infection, delaying optimal medication until the diagnosis is confirmed is not an ideal option, as trophozoites in cerebrospinal fluid are detected by time-consuming methods. In most of the presented cases, patients became comatose by the time of diagnostic confirmation and under correct therapy they decompensated [47]. The methods of choice for diagnosis are evaluation of the CSF smear and brain biopsy, but in recent years, there have been studies that have shown the efficiency and rapidity of next-generation sequencing (NGS) [58]. Even so, if the *Naegleria fowleri* infection is not taken into account and therapy is not initiated until the results are received, the chances of survival decrease, especially as there is currently no drug with substantial therapeutic efficacy, the mortality rate being approximately 98% [55].

### 4.3. Treatment

Regarding treatment, active substances proven to be useful include Amphotericin B, Miconazole, Tetracycline, and Rifampicin. The drug of choice used to treat this infection is Amphotericin B, administered intravenously and intrathecally, thus ensuring an increased concentration in the cerebrospinal fluid [4]. Lately, an antiparasitic called Miltefosine has been added to the basic therapeutic regimen, together with Amphotericin B, Rifampicin, and Fluconazole, with an increased success rate [56,57]. This was validated as a therapy in 2022 after an 8-year-old child was completely cured after it was added to the therapeutic regimen. Even though Amphotericin B has proven its efficacy, the similar adverse effects with patients’ onset symptoms plus nephrotoxicity emphasize the need for a new therapy. Moreover, Miltefosine has proven to be effective when it comes to patient survival following *Naegleria fowleri* infection, but in most cases, patients are neurologically affected [61].

Recently, there has been increasing discussion about new nanoparticle-based therapies, which are superior to older drugs because they can cross the blood–brain barrier much more easily, without the need for an additional dose increase [61,62]. Studies show that a large amount of the drug could be administered intranasally via these nanoparticles, but the cytotoxic effects and pharmacokinetics are not fully known [63]. For example, in 2017, a study of a silver nanoparticle conjugated with Amphotericin B, Nystatin, and Fluconazole was reported, which showed in vitro efficacy against the brain-eating amoeba but showed a cytotoxic effect of up to 75% [64]. On the other hand, the 50 µM concentration of a gold nanoparticle conjugated with trans-cinnamic acid showed efficacy as high as Amphotericin B, with no signs of cell toxicity [65].

## 5. Conclusions

The cases presented in this review are proof that delaying therapy until the diagnosis is confirmed or even because not considering *Naegleria fowleri* infection can lead to a fulminant evolution with an unfortunate outcome. It is important that physicians who see patients with meningitis-specific symptoms of any cause should also consider *Naegleria fowleri* infection, especially in young people with a history of warm-season swimming in unhygienic, inadequately irrigated places, as prompt initiation of specific therapy may increase survival rates.

## 6. Limitations

Considering that the cases described did not follow a unique writing protocol, the lack of standardization of case presentation may lead to incomplete information about the cases included in the review. Moreover, the small number of cases reported at the global level may lead to misleading general conclusions, especially regarding the appropriate treatment for this type of patient. Another limitation can be considered the lack of a uniform sample of patients, due to the small number of cases; thus, conclusions about the exact cause of infection may not be clear and accurate. Moreover, the reasons why some patients are more susceptible to *Naegleria fowleri* infection have not yet been presented in the literature, especially since most of the patients were infected through contact with contaminated water with which many other people had previously come into contact.

## Figures and Tables

**Table 1:** Characteristics of cases included in the review.

Author, and Year	Country	Number of Cases	Sex and Age	Causes	Onset Symptoms	Diagnostic Test	Medical History
J. Apley et al. [11]	UK	3 cases	Case 1—male, 2 years. Case 2—male, 6 years. Case 3—male, 4 years	Case 1–3—playing with contaminated water (muddied puddle)	C1–C3: anorexia, irritability, sore throat, vomiting, headache, fever, neck pains	Cultured/wet mount from cerebrospinal fluid (CSF)	C3: 2 days before admission, he had a booster dose of diphtheria pertussis and tetanus vaccine
A.R. Cain et al., 1981 [12]	UK	1 case	Female, 11 years	Contaminated pool water (indoor pool fed by natural warm spring water)	Headache Fever Vomiting Blurred vision	CSF	Healthy
AR Stevens et al., 1981 [13]	USA	2 cases	Case 1—male, 14 years Case 2—male, 10 years	Case 1 and 2—swimming in contaminated water (freshwater lake)	C1: headache, fever, malaise C2: headache, lethargy, anorexia	CSF	Healthy
N.D.P. Barnett et al., 1996 [14]	USA	2 case	Case 1—Female, 9 years Case 2—male, 8 month	Case 1—Swimming in contaminated water (ditch) Case 2—baptized in contaminated water	C1: Headache Emesis C2: emesis, fever	C1 and C2: cranial CT scan, CSF	Healthy
Y. Sugita et al., 1999 [15]	Japan	1 case	Female, 25 years	No data	Headache High fever	CSF, cranial CT scan	Healthy
Jain et al., 2002 [16]	India	1 case	Female, 26 years	No data	Headache Fever Vomiting Altered sensorium	CSF	Healthy
S Shenoy et al., 2002 [17]	India	1 case	Male, 5-month-old	Contaminated bath water	Fever Vomiting Seizures	CSF	Healthy
Centers for Disease Control and Prevention (CDC), 2003 [18]	USA	1 case	Male, 11 years	Swimming in contaminated water (local river)	Headache Emesis	CSF and cranial MRI	Healthy
P.E. Cogo et al., 2004 [19]	Italy	1 case	Male, 9 years	Swimming in contaminated river water	Fever Headache	CSF and cranial CT	Healthy
D.T. Okuda et al., 2004 [20]	USA	2 cases	Case 1—male, 5 years Case 2—male, 5 years	Case 1—no data Case 2—contaminated water (bath water)	C1: Headache, neck stiffness C2: fever, progressive lethargy	CSF and cranial MRI	Healthy
S. Hebbar et al., 2005 [21]	India	1 case	Male, 6 months	Contaminated bath water	Seizures Fever Lethargy Altered sensorium	CSF	Healthy
J. Vargas-Zepada el al, 2005 [22]	Mexic	1 case	Male, 10 years	Swimming in contaminated water (irrigation canal)	Severe headache Vomiting Fever	CSF, cranial CT	Healthy
F. Petit et al., 2006 [23]	Venezuela	2 cases	Case 1—male, 10 years Case 2—male, 23 years	Swimming in contaminated water reservoir	C1: Headache Fever Vomiting C2: headache, fever, vomiting, drowsiness, behavioral disturbances	CSF	Healthy
CDC, 2008 [24]	USA	6 cases	Case 1—male, 14 years Case 2—male, 14 years Case 3—male, 11 years Case 4—male, 12 years Case 5—male, 22 years Case 6—male, 10 years	Case 1, 3, 4, 5— swimming in contaminated lake water Case 2—swimming in multiple drainage ditches, canals, and apartment pool Case 6—swimming in a private water sports facility	C1: severe headache, stiff neck, fever C2: ear pressure, severe headache, vomiting C3: headache, fever, nausea, vomiting, confusion C4: fever, lethargy, confusion C5: altered mental status, severe headache C6: body aches, high fever, nausea, vomiting, fainting	CSF	Healthy
N. Gupta et al., 2009 [25]	India	1 case	Male, 20 years	No association with contaminated water	Fever Headache Loss of vision Hearing loss Slurring of speech Difficulty in swallowing Retention of urine	CSF, cranial CT scan	Tuberculosis, diabetes mellitus, hypertension, and acute leukemic leukemia.
T. Saleem et al., 2009 [26]	Pakistan	2 cases	Case 1—male, 24 years Case 2—male, 30 years	Case 1 and 2—swimming in contaminated water	C1: high fever, headache, vomiting C2: high fever, headache, agitation	CSF and cranial CT scan	Healthy
S. Shakoor et al., 2011 [27]	Pakistan	13 cases	12/13 were male, mean age 31.0 ± 15.33 years	No exact data	Fever Headache Seizures	CSF	Healthy
Khanna et al., 2011 [28]	India	1 case	Male, 5 months	No data	Fever Decreases breastfeeding Vomiting Abnormal body movements	CSF	Healthy
Gautam et al., 2012 [29]	India	1 case	Male, 73 years	No data	Fever Neck pain Seizures Altered sensorium	CSF and cranial CT scan	Type II diabetes mellitus, diabetic nephropathy, coronary artery disease (postangioplasty in 2005), head injury (9 years back), and CSF (cerebrospinal fluid) rhinorrhea
S.K. Kemble et al., 2012 [4]	USA	1 case	Female, 7 years	Swimming in contaminated lake water	Headache Abdominal pain Neck soreness	CSF, PCR, and CT scan	Healthy
J.S. Yoder et al., 2012 [30]	USA	2 cases	Case 1—male, 28 years Case 2—female, 51 years	Contaminated tap water utilized for sinus Irrigation	C1: severe headache, neck stiffness, back pain, vomiting C2: altered mental status, nausea, vomiting, poor appetite, fatigue, high fever	C1: CSF, cranial CT scan, PCR C2: CSF	C1: migraine C2: Healthy
Z. Movahedi et al., 2012 [31]	Iran	1 case	Male, 5-month-old	No data	Fever Eye gaze Chills	CSF and cranial CT scan	Healthy
CDC, 2013 [32]	USA	1 case	Male, 47 years	Contaminated tap water used for daily household activities and for ablution	Headache Fever Confusion Agitation	CSF and PCR	Healthy
M.Y. Su et al., 2013 [33]	Taiwan	1 case	Male, 75 years	Swimming in contaminated pool water (hot springs)	Headache Fever Right arm myoclonic seizures	CSF, MRI of the brain, cranial CT scan	Healthy
A. Sood et al., 2014 [34]	India	1 case	Male, 6 years	Playing with contaminated water (cement tank)	Fever Headache Altered sensorium	CSF	Healthy
A. Shariq et al., 2014 [35]	Pakistan	1 case	Male, 42 years	Using contaminated water	Fever Vomiting Loose stools Behavioral disturbances	CSF	Healthy
P.J. Booth et al., 2015 [36]	USA	1 case	Male, 11 years	Swimming in contaminated pool water (resort hot springs)	Headache Fever Stiff neck Nausea Vomiting	CSF	Healthy
J.R. Cope et al., 2015 [37]	USA	1 case	Male, 4 years	Playing with contaminated pool water	Vomiting Severe headache Diarrhea Poor oral intake	CSF and cranial CT scan	Healthy
R.O. Johnson et al., 2016 [38]	USA	1 case	Female, 21 years	Swimming in contaminated spring water	Headache Nausea Vomiting	CSF	Healthy
J.R. Cope et al., 2016 [39]	USA	2 cases	Case 1—male, 12 years Case 2—male, 8 years	Case 1—contaminated stagnant rainwater Case 2—contaminated river water	C1: headache, weakness, vomiting, fever, altered mental status C2: fever, headache, chills, nausea, vomiting, altered mental status	C1: CSF, cranial CT scan, PCR C2: CSF	Healthy
T.T. Stubhaug et al., 2016 [40]	Norway	1 case	Female, 71 years	Contaminated tap water	Nausea, vomiting, fever, exhaustion	CSF, cranial CT scan	Healthy
R.C. Stowe et al., 2017 [41]	USA	2 cases	Case 1—male, 5 years Case 2—male, 14 years	Case 1 and 2—swimming in contaminated water	C1: fever, altered mental status, seizures C2: generalized muscle weakness, fever	C1: CSF, cranial CT, brain MRI C2: CSF, cranial CT	Healthy
J.R. Cope et al., 2017 [42]	USA	1 case	Female, 18 years	Swimming in contaminated water	Headache Fever Lethargy	CSF, cranial CT scan	Healthy
T.W. Heggie et al., 2017 [43]	USA	1 case	Female, 12 years	Swimming in contaminated lake water	Vomiting Fever Headache	CSF	Healthy
N.K. Ghanchi et al., 2017 [44]	Pakistan	19 cases	84% male, median age 28 years (16/19)	No exact data	Fever (63%) Altered consciousness (53%) Headache (32%) Seizures (21) Disorientation (10%)	CSF and PCR	Healthy
M. Chomba et al., 2017 [45]	Zambia	1 case	Male, 24 years	Swimming in contaminated river water	Seizures Fever	CSF, cranial CT	Healthy
Q. Wang et al., 2018 [46]	China	1 case	Male, 42 years	Contaminated water	Fever Headache	CSF, cranial CT	Healthy
M. Chen et al., 2019 [47]	China	1 case	Male, 43 years	Swimming in contaminated pool water	Headache Fever Myalgia Fatigue	CSF, cranial CT	Healthy
A. McLaughlin et al., 2019 [48]	Australia	1 case	Male, 56 years	Swimming in contaminated water/irrigation of nostrils	Headache Photophobia Nausea Vomiting Neck stiffness	CSF, cranial CT	No data
L.R. Moreira et al., 2020 [49]	Costa Rica	3 cases	Case 1—male, 15 years Case 2—female, 5 years Case 3—male, 1 year	Case 1 and 2—contaminated pool water (hot spring resort) Case 3—contaminated bath water	C1: general discomfort, severe headache, nausea, vomiting C2: lower limb pain, spasticity, hyperreflexia, walking difficulty, headache, vomiting, fever C3: fever, drowsiness, altered state of consciousness.	C1: no data C2 and C3: CSF	Healthy
S. Huang et al., 2021 [50]	China	1 case	Male, 8 years	Swimming in contaminated water	Headache Vomiting Fever Disturbance of consciousness	CSF, cranial CT scan, head and neck MRI	No data
Y. Celik et al., 2021 [51]	Turkey	1 case	Male, 11 days old	Contaminated bath water	Irritability Inability to suck Fever	CSF, cranial MRI, PCR	Healthy
S.K. Anjum et al., 2021 [52]	USA	1 case	Male, 13 years	Swimming in contaminated water (water park)	Headache Fever Intractable emesis	CSF, cranial CT scan, brain MRI, PCR	History of headache
P. Soontrapa et al., 2022 [53]	Thailand	1 case	Female, 40 years	Contaminated water (she poured water from a waterfall on her head and face)	Severe headache High fever	CSF, cranial CT scan	No data
P. Maloney et al., 2022 [54]	USA	1 case	Male, 8 years	Swimming in contaminated river water	Fever Altered mental status Malaise Headache Fatigue Decreased appetite	CSF, cranial CT scan	Healthy
X. Che et al., 2023 [55]	China	1 case	Male, 38 years	No exact data	Fever Headache Disturbance of consciousness	CSF and cranial CT scan	No data
K.W. Hong et al., 2023 [8]	Korea	1 case	Male, 52 years	No exact data	Headache Fever	CSF and cranial CT scan	No data
N.N. Baqer et al., 2023 [2]	Iraq	1 case	Female, 18 years	Contaminated river water	Fever Headache Stiff neck	CSF, PCR, cranial CT scan	Weight-loss and malnutrition
F. Wang et al., 2023 [56]	China	1 case	Male, 62 years	Contaminated water (the patient was a fisherman)	Vomiting Headache Behavior change	CSF and cranial CT scan	No data
Q. Wu et al., 2024 [57]	China	1 case	Male, 42 years	He drank tea and was washed by his mother with spring water.	High fever	CSF, cranial CT scan	The patient was bed-ridden due to a disability caused by burns
L. Lin et al., 2024 [58]	China	1 case	Female, 6 year	Swimming in contaminated pool water	Fever Headache Vomiting Lethargy	Metagenomic next-generation sequencing, CSF, PCR, cranial CT scan	Healthy
S.N. Puthanpurayil et al., 2024 [59]	India	1 case	Male, 36 years	Contaminated tap water (nasal irrigation)	Seizures Altered sensorium Headache Nausea Photophobia High fever	CSF	Healthy, 15-year-old corrected surgically nasal bone fracture

Blood samples for biochemistry and hemogram were taken from all patients.

**Table 2:** Evolution and treatment of the patients included in the review.

Author	Timeline Between Exposure and Onset of Symptoms	Timeline Between Onset of Symptoms and Presentation to Hospital	Antibiotic Treatment	Days of Hospitalization	Evolution
J. Apley et al. [11]	C1: 2 days C2: 9 days C3: 13 days	C1: One day before admission C2: on the morning of admission C3: on the morning of admission	Case 1—Sulfadiazine, Penicilina, Ampicilina, Amphotericin B Case 2—Sulfadiazine, Amphotericin B Case 3—Sulfadiazine, Amphotericin B Amphotericin 0.25 mg/kg/day iv. to 1 mg/kg/day iv Sulphadiazine 750 mg/6 h	Case 1—16 days; Case 2—18 days; Case 3—24 days	Case 1—died; Case 2—cured; Case 3—cured
A.R. Cain et al. [12]	6 days	3 days	Penicillin, Sulfadimidine, Chloramphenicol, Sulfadiazine, Metronidazole, Amphotericin B. Amphotericin B 0.5 mg to 0.6 mg/kg/day/iv Amphotericin B 0.1 mg through an intraventricular catheter	4 days	Died
A.R. Stevens et al. [13]	C1: 3 weeks before C2: several days before	C1: 2 days C2: on the day of admission into the hospital	Case 1—Penicillin G 3 g/4 h, Amphotericin B 10 mg/day iv and 0.05 mg/day intrathecally, Miconazole 200 mg/day iv and intrathecally 20 mg/day Case 2—Penicillin 1.25 million units/6 h iv, Amphotericin B 1 mg/kg/day iv and intraventricular 0.1 mg	Case 1 and 2—5 days	Case 1 and 2—died
N.D.P. Barnett et al. [14]	C1: The day after exposure C2: soon after baptized	C1: 2 days C2: 24 h	C1 and C2: Cefuroxime, Acyclovir No data regarding doses	C1: 4 days C2: 3 days	C1 and C2: Died
Y. Sugita et al. [15]	No data	2 days	No data	8 days	Died
Jain et al. [16]	No data	10 days	Amphotericin B 1 mg/kg/day iv Rifampicin 450 mg/day po Ornidazole 500 mg/8 h	28 days	Cured
S. Shenoy et al. [17]	No data	1 week	Amphotericin B 0.6 mg/kg/day iv Ceftriaxone 100 mg/kg/day po	2 days	Died
Centers for Disease Control and Prevention (CDC) [18]	No data	2 days	Amphotericin B, Rifampicin, Ketoconazole	4 days	Died
P.E. Cogo et al. [19]	10 days	1 day	Ceftriaxone, Acyclovir 0.35 g/kg/6 h	4 days	Died
D.T. Okuda et al. [20]	No data	C1: no data C2: 3 days	Case 1—no data Case 2—empiric antibiotic	Case 1—48 h Case 2—no data	Case 1—died Case 2—died
S. Hebbar et al. [21]	No data	3 days	Amphotericin B, Chloramphenicol and Metronidazole	10 h	Died
J. Vargas-Zepada et al. [22]	One week before admission into the hospital	On the day of admission into the hospital	Ceftriaxone 100 mg/kg/8 h iv, Rifampicin 10 mg/kg/24 h po and Amphotericin B 0.25 mg/kg/24 h iv (daily 0.25 mg/kg increasing dosage up to 1 mg/kg/day), Fluconazole 10 mg/kg/24 h iv	23 days	Cured
F. Petit et al. [23]	C1: 5 days C2: no data	C1: one day before admission into the hospital C2: 4 days before admission into the hospital	Case 1—Amphotericin B Case 2—no data	Case 1—3 days Case 2—2 days	Case 1—died Case 1—died
CDC [24]	C1: 7 days C2: 2 weeks C3: 6 days C4: weeks C5: 7 days C6: 8–15 days	C1: 2 days C2: 2 days C3: 4 days C4: 6 days C5: 2 days C6: 3 days	Case 1—no data Case 2—no data Case 3—Amphotericin B, Fluconazole, Ceftriaxone, Azithromycin, Rifampicin Case 4—Amphotericin B, Rifampicin, Azithromycin Case 5—no data Case 6—Amphotericin B, Rifampicin, Azithromycin, Fluconazole	Case 1—2 days Case 2—no data Case 3—3 days Case 4—5 days Case 5—5 days Case 6—3 days	Case 1—died Case 2—died Case 3—died Case 4—died Case 5—died Case 6—died
N Gupta et al. [25]	No data	2 days	Ceftriaxone, Amikacin, Amphotericin B, Rifampicin	No exact data	Died
T. Saleem et al. [26]	No data	C1: 2 days C2: 3 days	Case 1—Ceftriaxone, Acyclovir, Vancomycin, Meropenem, Amphotericin B, Fluconazole, Rifampicin Case 2—Acyclovir, Ceftriaxone, Meropenem, Vancomycin, Amphotericin B, Fluconazole	Case 1—6 days Case 2—8 days	Case 1—died Case 2—died
S. Shakoor et al. [27]	No data	Mean ±SD: 2.5 ± 1.19 days	Amphotericin B (1.5 mg/kg/day/iv), Rifampin (600 mg/day), Fluconazole or Itraconazole	6.38 ± 3.15 days	Died
Khanna et al. [28]	No data	2 days	Ceftriaxone 250 mg TID and iv, Amikacin 50 mg BD Amphotericin B 3 mg iv, ceftazidime 300 mg iv	2 days	Died
Gautam et al. [29]	No data	3 days	Amphotericin B (1 mg/kg/day) and oral Rifampicin (600 mg OD)	10 days	Cured
S.K. Kemble et al. [4]	2 weeks	5 days	Penicillin, Ceftriaxone, Vancomycin	4 days	Died
J.S. Yoder et al. [30]	No data	C1: 1 day C2: 3 days	Case 1—Ceftriaxone, Linezolid, Acyclovir, Amphotericin B, Rifampin Case 2—no data	Case 1—4 days Case 2—5 days	Case 1—died Case 2—died
Z. Movahedi et al. [31]	No data	3 days	Rifampin 10 mg/kg orally daily, Amphotericin B 1 mg/kg/day, Ceftriaxone, Vancomycin	No exact data	Cured
CDC [32]	No exact data	No exact data	No data	34 days	Died
M.Y. Su et al. [33]	No data	No data	Intravenous amphotericin B 50 mg/day	21 days	Died
A. Sood et al. [34]	No data	No data	Intravenous amphotericin B (1 mg/kg), intravenous Fluconazole (8 mg/kg), and oral Rifampicin (10 mg/kg) for 21 days	21 days	Cured
A. Shariq et al. [35]	No data	2 days	Amphotericin-B 1.5 mg/kg iv divided in two divided doses daily plus 1.5 mg/day intrathecal	3 days	Died
P.J. Booth et al. [36]	4 days	2 days	No data	4 days	Died
J.R. Cope et al. [37]	10 days	1 day	Vancomycin, Ceftriaxone	5 days	Died
R.O. Johnson et al. [38]	2 weeks	1 day	No data	3 days	Died
J.R. Cope et al. [39]	No data	C1: 2 days C2: 5 days	Case 1—Acyclovir, Amphotericin B, Fluconazole, Rifampin, Vancomycin, Ceftriaxone, Azithromycin, Miltefosine Case 2—Miltefosine	Case 1—16 days Case 2—85 days	Case 1—died Case 2—cured, but remains with profound persistent mental disability
T.T. Stubhaug et al. [40]	Approx. 12 days	2 days	Meropenem, Vancomicin, Gentamicin	No exact data	Died
R.C. Stowe et al. [41]	C1: 8 days C2: 8 days	C1: 5 days C2: 3 days	Case 1—Azithromycin, Rifampin, Amphotericin B, Fluconazole, Miltefosine Case 2—Vancomycin, Ceftriaxone, Fluconazole, Azithromycin, Rifampin, Amphotericin, Miltefosine	Case 1—2 days Case 2—4 days	Case 1—died Case 2—died
J.R. Cope et al. [42]	Approx. 10 days	3 days	Amphotericin B, Fluconazole, Azithromycin, Rifampin	3 days	Died
T.W. Heggie et al. [43]	Few days	2 days	Amphotericin B, Rifampin, Fluconazole, Dexamethasone, Azithromycin, Miltefosine	55 days	Cured
N.K. Ghanchi et al., 2017 [44]	No data	2–3 days	Therapeutic protocol for primary amoebic meningoencephalitis	3–4 days	18/19 died
M. Chomba et al., 2017 [45]	2 days	1 day	Amphotericin B 50 mg IV, Ceftriaxone 2 g/day iv	8 days	Died
Q. Wang et al., 2018 [46]	1 week	1 day	Ceftriaxone 2 g, Meropenem, Linezolid, Amphotericin B at 50 mg/day, Fluconazole 0.4 g/day	15 days	Died
M. Chen et al. [47]	No exact data	2 days	Amphotericin B, Fluconazole	15 days	Died
A. McLaughlin et al. [48]	No exact data	36 h	Intrathecal amphotericin 1.5 mg daily, amphotericin 50 mg/12 h IV, Rifampicin 600 mg IV daily, azithromycin 500 mg IV daily, Fluconazole 800 mg IV daily	3 days	Died
L.R. Moreira et al. [49]	C1: 7 days C2: 2 days C3: no exact data	C1: no data C2: 1 day C3: 3 days	Case 1—no data Case 2—Amphotericin B Case 3—no data	Case 1—6 days Case 2—28 days Case 3—1 day	Case 1—died Case 2—cured Case 3—died
S. Huang et al. [50]	3 days	1 day	Meropenem, Vancomycin, Ceftriaxone	24 days	Died
Y. Celik et al. [51]	4 days	2 days	Ampicillin, Cefotaxime, Vancomycin, Meropenem, Amphotericin B, Fluconazole, Rifampicin, Azithromycin	Approx. 4 months	Died
S.K. Anjum et al. [52]	3 days	On the day of admission into the hospital	Ceftriaxone, Acyclovir, Vancomycin, Miltefosine, Amphotericin B, Fluconazole, Rifampin, Azithromycin	5 days	Died
P. Soontrapa et al. [53]	3 days	1 day	Ceftriaxone, Doxycycline, Amphotericin B, Rifampicin, Fluconazole, Azithromycin	5 days	Died
P. Maloney et al. [54]	5 days	3 days	Amphotericin B, Azithromycin, Fluconazole, Rifampin	28 h	Died
X. Che et al. [55]	No data	2 days	Penicillin, Ceftriaxone	4 days	Died
K.W. Hong et al. [8]	No exact data	3 days	Vancomycin, Ceftriaxone, Ampicillin, Amphotericin B, Fluconazole, Azithromycin, Rifampicin	13 days	Died
N.N. Baqer et al. [2]	No exact data	2 days	No treatment	No data	Died
F. Wang et al. [56]	No exact data	3 days	No exact data	3 days	Died
Q. Wu et al. [57]	Approx. 1 week	No data	Meropenem (2000 mg/8 h), Metronidazole (500 mg/8 h), Fluconazole (800 mg/day), Piperacillin-Tazobactam	2 days	Died
L Lin et al. [58]	7 days	14 h	Cefaclor, Meropenem, Acyclovir, Vancomycin, Amphotericin B, Rifampicin	80 h	Died
S.N. Puthanpurayil et al. [59]	No data	2 days	Ceftriaxone Acyclovir	No exact data	Died

## References

[1] Matin A. (2017). Primary amebic meningoencephalitis: A new emerging public health threat by *Naegleria fowleri* in Pakistan. J. Pharm. Res. Drug Des..

[2] Baqer N.N., Mohammed A.S., Al-Aboody B., Ismail A.M. (2023). Genetic Detection of Amoebic Meningoencephalitis Causing by Naegleria Fowleri in Iraq: A Case Report. Iran J. Parasitol..

[3] Hall A.D., Kumar J.E., Golba C.E., Luckett K.M., Bryant W.K. (2024). Primary amebic meningoencephalitis: A review of *Naegleria fowleri* and analysis of successfully treated cases. Parasitol. Res..

[4] Kemble S.K., Lynfield R., DeVries A.S., Drehner D.M., Pomputius W.F., Beach M.J., Visvesvara G.S., da Silva A.J., Hill V.R., Yoder J.S. (2012). Fatal Naegleria fowleri infection acquired in Minnesota: Possible expanded range of a deadly thermophilic organism. Clin. Infect. Dis..

[5] Zeibig E. (2013). Clinical Parasitology: A Practical Approach.

[6] Mahmud R., Ai Lian Lim Y., Amir A. (2017). Medical Parasitology: A Textbook.

[7] Radulescu S. (2000). Parazitologie Medicala.

[8] Hong K.W., Jeong J.H., Byun J.H., Hong S.H., Ju J.W., Bae I.G. (2023). Fatal Primary Amebic Meningoencephalitis due to *Naegleria fowleri*: The First Imported Case in Korea. Yonsei Med. J..

[9] Gharpure R., Bliton J., Goodman A., Ali I.K.M., Yoder J., Cope J.R. (2021). Epidemiology and Clinical Characteristics of Primary Amebic Meningoencephalitis Caused by *Naegleria fowleri*: A Global Review. Clin. Infect. Dis..

[10] Saberi R., Seifi Z., Dodangeh S., Najafi A., Abdollah Hosseini S., Anvari D., Taghipour A., Norouzi M., Niyyati M. (2020). A systematic literature review and meta-analysis on the global prevalence of *Naegleria* spp. in water sources. Transbound. Emerg. Dis..

[11] Apley J., Clarke S.K., Roome A.P., Sandry S.A., Saygi G., Silk B., Warhurst D.C. (1970). Primary amoebic meningoencephalitis in Britain. Br. Med. J..

[12] Cain A.R., Wiley P.F., Brownell B., Warhurst D.C. (1981). Primary amoebic meningoencephalitis. Arch. Dis. Child..

[13] Stevens A.R., Shulman S.T., Lansen T.A., Cichon M.J., Willaert E. (1981). Primary amoebic meningoencephalitis: A report of two cases and antibiotic and immunologic studies. J. Infect. Dis..

[14] Barnett N.D., Kaplan A.M., Hopkin R.J., Saubolle M.A., Rudinsky M.F. (1996). Primary amoebic meningoencephalitis with Naegleria fowleri: Clinical review. Pediatr. Neurol..

[15] Sugita Y., Fujii T., Hayashi I., Aoki T., Yokoyama T., Morimatsu M., Fukuma T., Takamiya Y. (1999). Primary amebic meningoencephalitis due to *Naegleria fowleri*: An autopsy case in Japan. Pathol. Int..

[16] Jain R., Prabhakar S., Modi M., Bhatia R., Sehgal R. (2002). Naegleria meningitis: A rare survival. Neurol. India.

[17] Shenoy S., Wilson G., Prashanth H.V., Vidyalakshmi K., Dhanashree B., Bharath R. (2002). Primary meningoencephalitis by Naegleria fowleri: First reported case from Mangalore, South India. J. Clin. Microbiol..

[18] Centers for Disease Control and Prevention (CDC) (2003). Primary amebic meningoencephalitis-Georgia, 2002. Morb. Mortal Wkly. Rep. MMWR.

[19] Cogo P.E., Scagli M., Gatti S., Rossetti F., Alaggio R., Laverda A.M., Zhou L., Xiao L., Visvesvara G.S. (2004). Fatal Naegleria fowleri meningoencephalitis, Italy. Emerg. Infect. Dis..

[20] Okuda D.T., Hanna H.J., Coons S.W., Bodensteiner J.B. (2004). Naegleria fowleri hemorrhagic meningoencephalitis: Report of two fatalities in children. J. Child. Neurol..

[21] Hebbar S., Bairy I., Bhaskaranand N., Upadhyaya S., Sarma M.S., Shetty A.K. (2005). Fatal case of Naegleria fowleri meningo-encephalitis in an infant: Case report. Ann. Trop. Paediatr..

[22] Vargas-Zepeda J., Gómez-Alcalá A.V., Vásquez-Morales J.A., Licea-Amaya L., De Jonckheere J.F., Lares-Villa F. (2005). Successful treatment of Naegleria fowleri meningoencephalitis by using intravenous amphotericin B, fluconazole and rifampicin. Arch. Med. Res..

[23] Petit F., Vilchez V., Torres G., Molina O., Dorfman S., Mora E., Cardozo J. (2006). Meningoencefalitis amebiana primaria: Comunicacion de dos nuevos casos Venezolanos. Arq. de Neuro-Psiquiatria.

[24] Centers for Disease Control and Prevention (CDC) (2008). Primary amebic meningoencephalitis-Arizona, Florida, and Texas, 2007. Morb. Mortal Wkly. Rep. MMWR.

[25] Gupta N., Bhaskar H., Duggal S., Ghalaut P.S., Kundra S., Arora D.R. (2009). Primary amoebic meningoencephalitis: First reported case from Rohtak, North India. Braz. J. Infect. Dis..

[26] Saleem T., Rabbani M., Jamil B. (2009). Primary amoebic meningoencephalitis: Two new cases from Pakistan. Trop. Doct..

[27] Shakoor S., Beg M.A., Mahmood S.F., Bandea R., Sriram R., Noman F., Ali F., Visvesvara G.S., Zafar A. (2011). Primary amebic meningoencephalitis caused by Naegleria fowleri, Karachi, Pakistan. Emerg. Infect. Dis..

[28] Khanna V., Khanna R., Hebbar S., Shashidhar V., Mundkar S., Munim F., Annamalai K., Nayak D., Mukhopadhayay C. (2011). Primary Amoebic Meningoencephalitis in an Infant due to Naegleria fowleri. Case Rep. Neurol. Med..

[29] Gautam P.L., Sharma S., Puri S., Kumar R., Midha V., Bansal R. (2012). A rare case of survival from primary amebic meningoencephalitis. Indian J. Crit. Care Med..

[30] Yoder J.S., Straif-Bourgeois S., Roy S.L., Moore T.A., Visvesvara G.S., Ratard R.C., Hill V.R., Wilson J.D., Linscott A.J., Crager R. (2012). Primary amebic meningoencephalitis deaths associated with sinus irrigation using contaminated tap water. Clin. Infect. Dis..

[31] Movahedi Z., Shokrollahi M.R., Aghaali M., Heydari H. (2012). Primary amoebic meningoencephalitis in an Iranian infant. Case Rep. Med..

[32] Centers for Disease Control and Prevention (CDC) (2013). Notes from the field: Primary amebic meningoencephalitis associated with ritual nasal rinsing-St. Thomas, U.S. Virgin Islands, 2012. Morb. Mortal Wkly. Rep. MMWR.

[33] Su M.Y., Lee M.S., Shyu L.Y., Lin W.C., Hsiao P.C., Wang C.P., Ji D.D., Chen K.M., Lai S.C. (2013). A fatal case of Naegleria fowleri meningoencephalitis in Taiwan. Korean J. Parasitol..

[34] Sood A., Chauhan S., Chandel L., Jaryal S.C. (2014). Prompt diagnosis and extraordinary survival from Naegleria fowleri meningitis: A rare case report. Indian J. Med. Microbiol..

[35] Shariq A., Afridi F.I., Farooqi B.J., Ahmed S., Hussain A. (2014). Fatal primary meningoencephalitis caused by Naegleria fowleri. J. Coll. Physicians Surg. Pak..

[36] Booth P.J., Bodager D., Slade T.A., Jett S. (2015). Primary Amebic Meningoencephalitis Associated with Hot Spring Exposure During International Travel—Seminole County, Florida, July 2014. Morb. Mortal Wkly. Rep. MMWR.

[37] Cope J.R., Ratard R.C., Hill V.R., Sokol T., Causey J.J., Yoder J.S., Mirani G., Mull B., Mukerjee K.A., Narayanan J. (2015). The first association of a primary amebic meningoencephalitis death with culturable Naegleria fowleri in tap water from a US treated public drinking water system. Clin. Infect. Dis..

[38] Johnson R.O., Cope J.R., Moskowitz M., Kahler A., Hill V., Behrendt K., Molina L., Fullerton K.E., Beach M.J. (2016). Notes from the Field: Primary Amebic Meningoencephalitis Associated with Exposure to Swimming Pool Water Supplied by an Overland Pipe—Inyo County, California, 2015. Morb. Mortal Wkly. Rep. MMWR.

[39] Cope J.R., Conrad D.A., Cohen N., Cotilla M., DaSilva A., Jackson J., Visvesvara G.S. (2016). Use of the Novel Therapeutic Agent Miltefosine for the Treatment of Primary Amebic Meningoencephalitis: Report of 1 Fatal and 1 Surviving Case. Clin. Infect. Dis..

[40] Stubhaug T.T., Reiakvam O.M., Stensvold C.R., Hermansen N.O., Holberg-Petersen M., Antal E.A., Gaustad K., Førde I.S., Heger B. (2016). Fatal primary amoebic meningoencephalitis in a Norwegian tourist returning from Thailand. JMM Case Rep..

[41] Stowe R.C., Pehlivan D., Friederich K.E., Lopez M.A., DiCarlo S.M., Boerwinkle V.L. (2017). Primary Amebic Meningoencephalitis in Children: A Report of Two Fatal Cases and Review of the Literature. Pediatr. Neurol..

[42] Cope J.R., Murphy J., Kahler A., Gorbett D.G., Ali I., Taylor B., Corbitt L., Roy S., Lee N., Roellig D. (2018). Primary Amebic Meningoencephalitis Associated with Rafting on an Artificial Whitewater River: Case Report and Environmental Investigation. Clin. Infect. Dis..

[43] Heggie T.W., Küpper T. (2017). Surviving Naegleria fowleri infections: A successful case report and novel therapeutic approach. Travel. Med. Infect. Dis..

[44] Ghanchi N.K., Jamil B., Khan E., Ansar Z., Samreen A., Zafar A., Hasan Z. (2017). Case Series of Naegleria fowleri Primary Ameobic Meningoencephalitis from Karachi, Pakistan. Am. J. Trop. Med. Hyg..

[45] Chomba M., Mucheleng’anga L.A., Fwoloshi S., Ngulube J., Mutengo M.M. (2017). A case report: Primary amoebic meningoencephalitis in a young Zambian adult. BMC Infect. Dis..

[46] Wang Q., Li J., Ji J., Yang L., Chen L., Zhou R., Yang Y., Zheng H., Yuan J., Li L. (2018). A case of Naegleria fowleri related primary amoebic meningoencephalitis in China diagnosed by next-generation sequencing. BMC Infect. Dis..

[47] Chen M., Ruan W., Zhang L., Hu B., Yang X. (2019). Primary Amebic Meningoencephalitis: A Case Report. Korean J. Parasitol..

[48] McLaughlin A., O’Gorman T. (2019). A local case of fulminant primary amoebic meningoencephalitis due to Naegleria fowleri. Rural Remote Health.

[49] Retana Moreira L., Zamora Rojas L., Grijalba Murillo M., Molina Castro S.E., Abrahams Sandí E. (2020). Primary Amebic Meningoencephalitis Related to Groundwater in Costa Rica: Diagnostic Confirmation of Three Cases and Environmental Investigation. Pathogens.

[50] Huang S., Liang X., Han Y., Zhang Y., Li X., Yang Z. (2021). A pediatric case of primary amoebic meningoencephalitis due to Naegleria fowleri diagnosed by next-generation sequencing of cerebrospinal fluid and blood samples. BMC Infect. Dis..

[51] Celik Y., Arslankoylu A.E. (2021). A Newborn with Brain-Eating Ameba Infection. J. Trop. Pediatr..

[52] Anjum S.K., Mangrola K., Fitzpatrick G., Stockdale K., Matthias L., Ali I.K.M., Cope J.R., O’Laughlin K., Collins S., Beal S.G. (2021). A case report of primary amebic meningoencephalitis in North Florida. ID Cases.

[53] Soontrapa P., Jitmuang A., Ruenchit P., Tiewcharoen S., Sarasombath P.T., Rattanabannakit C. (2022). The First Molecular Genotyping of Naegleria fowleri Causing Primary Amebic Meningoencephalitis in Thailand with Epidemiology and Clinical Case Reviews. Front. Cell Infect. Microbiol..

[54] Maloney P., Mowrer C., Jansen L., Karre T., Bedrnicek J., Obaro S.K., Iwen P.C., McCutchen E., Wetzel C., Frederick J. (2023). Fatal Primary Amebic Meningoencephalitis in Nebraska: Case Report and Environmental Investigation, August 2022. Am. J. Trop. Med. Hyg..

[55] Che X., He Z., Tung T.H., Xia H., Lu Z. (2023). Diagnosis of primary amoebic meningoencephalitis by metagenomic next-generation sequencing: A case report. Open Life Sci..

[56] Wang F., Shen F., Dai W., Zhao J., Chen X., Liu J. (2023). A primary amoebic meningoencephalitis case associated with swimming in seawater. Parasitol. Res..

[57] Wu Q., Chen C., Li J., Lian X. (2024). Primary Amebic Meningoencephalitis Caused by Naegleria fowleri in China: A Case Report. Infect. Microbes. Dis..

[58] Lin L., Luo L., Wu M., Chen J., Liao Y., Zhang H. (2024). Utilizing metagenomic next-generation sequencing and phylogenetic analysis to identify a rare pediatric case of *Naegleria fowleri* infection presenting with fulminant myocarditis. Front. Microbiol..

[59] Puthanpurayil S.N.T., Mukundan A., Nair S.R., John A.P., Thampi M.R., John R., Sehgal R. (2024). Free-living amoebic encephalitis—Case series. Trop. Parasitol..

[60] Lekkla A., Sutthikornchai C., Bovornkitti S., Sukthana Y. (2005). Free-living ameba contamination in natural hot springs in Thailand. Southeast Asian J. Trop. Med. Public Health.

[61] Güémez A., García E. (2021). Primary Amoebic Meningoencephalitis by Naegleria fowleri: Pathogenesis and Treatments. Biomolecules.

[62] Rajendran K., Anwar A., Khan N.A., Aslam Z., Raza Shah M., Siddiqui R. (2020). Oleic Acid Coated Silver Nanoparticles Showed Better in Vitro Amoebicidal Effects against Naegleria fowleri than Amphotericin B. ACS Chem. Neurosci..

[63] Fong H., Leid Z.H., Debnath A. (2024). Approaches for Targeting Naegleria fowleri Using Nanoparticles and Artificial Peptides. Pathogens.

[64] Rajendran K., Anwar A., Khan N.A., Siddiqui R. (2017). Brain-Eating Amoebae: Silver Nanoparticle Conjugation Enhanced Efficacy of Anti-Amoebic Drugs against Naegleria fowleri. ACS Chem. Neurosci..

[65] Rajendran K., Anwar A., Khan N.A., Shah M.R., Siddiqui R. (2019). trans-Cinnamic Acid Conjugated Gold Nanoparticles as Potent Therapeutics against Brain-Eating Amoeba *Naegleria fowleri*. ACS Chem. Neurosci..

